# Improving the UNC Passive Aerosol Sampler Model Based on Comparison with Commonly Used Aerosol Sampling Methods

**DOI:** 10.1093/annweh/wxx110

**Published:** 2017-12-28

**Authors:** Mariam Shirdel, Britt M Andersson, Ingvar A Bergdahl, Johan N Sommar, Håkan Wingfors, Ingrid E Liljelind

**Affiliations:** 1 Occupational and Environmental Medicine, Department of Public Health and Clinical Medicine, Umeå University, Umeå, Sweden; 2 Department of Applied Physics and Electronics, Umeå University, Umeå, Sweden; 3 CBRN Defence & Security Division, Swedish Defence Research Agency, Cementvägen, Umeå, Sweden

**Keywords:** inorganic dust, mesh factor, PM_10_, PM_2.5_, respirable fraction, UNC passive aerosol sampler, working environment

## Abstract

**Objectives:**

In an occupational environment, passive sampling could be an alternative to active sampling with pumps for sampling of dust. One passive sampler is the University of North Carolina passive aerosol sampler (UNC sampler). It is often analysed by microscopic imaging. Promising results have been shown for particles above 2.5 µm, but indicate large underestimations for PM_2.5_. The aim of this study was to evaluate, and possibly improve, the UNC sampler for stationary sampling in a working environment.

**Methods:**

Sampling was carried out at 8-h intervals during 24 h in four locations in an open pit mine with UNC samplers, respirable cyclones, PM_10_ and PM_2.5_ impactors, and an aerodynamic particle sizer (APS). The wind was minimal. For quantification, two modifications of the UNC sampler analysis model, UNC sampler with hybrid model and UNC sampler with area factor, were compared with the original one, UNC sampler with mesh factor derived from wind tunnel experiments. The effect of increased resolution for the microscopic imaging was examined.

**Results:**

Use of the area factor and a higher resolution eliminated the underestimation for PM_10_ and PM_2.5_. The model with area factor had the overall lowest deviation versus the impactor and the cyclone. The intraclass correlation (ICC) showed that the UNC sampler had a higher precision and better ability to distinguish between different exposure levels compared to the cyclone (ICC: 0.51 versus 0.24), but lower precision compared to the impactor (PM_10_: 0.79 versus 0.99; PM_2.5_: 0.30 versus 0.45). The particle size distributions as calculated from the different UNC sampler analysis models were visually compared with the distributions determined by APS. The distributions were obviously different when the UNC sampler with mesh factor was used but came to a reasonable agreement when the area factor was used.

**Conclusions:**

High resolution combined with a factor based on area only, results in no underestimation of small particles compared to impactors and cyclones and a better agreement with the APS’s particle size distributions. The UNC sampler had lower precision than the impactors, but higher than the respirable cyclone. The UNC sampler with area factor could be used for PM_2.5_, PM_10_ and respirable fraction measurements in this working environment without wind.

## Introduction

Measurements of the concentration of particulate matter in the occupational environment have mostly been done using active sampling by pumping air through a size-selective device followed by gravimetric determination (NIOSH, 1998). An active sampler needs to be calibrated and maintained and is difficult to manage for both area sampling and personal sampling. It can be perceived as bulky and heavy by occupational hygienists and the person wearing it. The measurements are time consuming and accompanied by considerable labour costs. Passive sampling may be an alternative as it is simple, cost-effective and less intrusive (Nothstein *et al.*, 2000).

Wagner and Leith (2001b) have presented the UNC passive aerosol sampler (UNC sampler), weighing 1.7 g, having a diameter of 15 mm. It is inexpensive and a good candidate for sampling, but has mainly been used for ambient sampling and very little in occupational environments. The UNC sampler collects particles by gravity, impaction and diffusion, following theories of particle deposition velocities according to particle size (Wagner and Leith, 2001a, b, c; Wagner and Macher, 2003; Leith *et al.*, 2007; Whitehead and Leith, 2008; Arashiro and Leith, 2012; Peters *et al.*, 2016). It consists of a flat circular aluminium plate in the form of a scanning electron microscope (SEM) stub. Attached onto the plate is a substrate (the collection surface), which is covered by a metal mesh cap to prevent deposition of large particles. The particles deposited on the collection surface are quantified by e.g. SEM, but other techniques may also be used to analyse the particles (Whitehead and Leith, 2008; Ott *et al.*, 2008; Wagner and Casuccio, 2014). The UNC sampler has previously shown results comparable to active samplers in laboratory environments and ambient environments, although it has not yet been extensively evaluated in working environments (Wagner and Leith, 2001a, b, c; Wagner and Macher, 2003; Leith *et al.*, 2007; Whitehead and Leith, 2008).

In a pilot study, the UNC sampler’s performance was compared to impactors in a mine (Shirdel *et al.*, 2017). It showed promising results for particles above 2.5 µm, but indicated large underestimations for PM_2.5_. Varying degrees of underestimations for PM_2.5_ have also previously been reported by Wagner and Macher (2003), Watkins *et al.* (2009), and Wagner *et al.* (2012).

Commonly studied particle size fractions are PM_10_, PM_2.5_, and respirable fraction. PM_10_ and PM_2.5_ have previously been investigated by the UNC sampler (Wagner and Leith, 2001a, 2001c; Leith *et al.*, 2007; Whitehead and Leith, 2008), while respirable fraction measurements with the UNC sampler have only been reported once (Wagner and Macher, 2003), but it is commonly used in occupational hygiene and is one of the particle fractions for which there are occupational exposure limits (OELs).

The aim of this study was to evaluate and possibly improve the UNC sampler for stationary sampling in the working environment of an open pit mine, for different particle size fractions and concentration ranges. The measured particle concentration of the UNC sampler was compared to particle concentrations of established active sampler methods; impactors (PM_10_, PM_2.5_) and cyclone (respirable fraction). The variability of the passive and active samplers was examined and the particle size distribution of the UNC sampler was related to those measured by an aerodynamic particle sizer (APS).

## Methods

### Sampling

Four locations were chosen for the stationary sampling; one outdoors: the crushing station; and three indoors: the drive station, the concentrator, and the concentrate terminal. It snowed during most of the sampling period. At each location, a 24-h period was divided into three 8 h intervals, following the workplace-specific shifts. Ten UNC samplers, three PM_10_ impactors, three PM_2.5_ impactors, three respirable cyclones, one field blank for each type of collection method and an APS with one sample continuously collected every 20 seconds were used for each 8-h interval. All samplers were placed near each other within an area of approximately 4 m^2^, and at the same height from the ground (1.5 ± 0.5 m).

The four locations were chosen to represent different environments that the workers are exposed to, as well as different characteristics in terms of particle size fractions and concentration range. The crushing station is located at 30 m below ground level in the open pit. After the ore is crushed it is transported by conveyor belts away from the crushing station and through the drive station towards the concentrator. When the ore reaches the mills at the concentrator, the ore is ground with water before being purified and dewatered. The subsequent copper concentrate, the purest form of the ore, is then transported to the concentrate terminal from where it is loaded onto rail wagons.

The temperature was measured at 5 min intervals with three ACR SmartButtons. The humidity and wind speed were measured at 20 min intervals with a simple weather station (Väderstation med pekskärm, 36–3242, Clas Ohlson, Sweden) containing a hygrometer and a cup anemometer.

### Passive methods

The stationary setup for the UNC samplers consisted of three shelters (RJ Lee Group, Monroeville, PA, USA), in the form of flat plates, as described by Ott and Peters (2008). In each of two shelters, three UNC samplers were placed, and four samplers in the last shelter. Electrically conductive copper tape, connected to the top, bottom and inside the mounting holes for the UNC samplers, were used for grounding the UNC sampler. The setup and samplers were grounded in accordance with Wagner and Leith (2001b) to limit the influence of static electricity.

Each UNC sampler consisted of an aluminium SEM stub (Ted Pella, Inc., Redding, CA, USA), a 12 mm leit adhesive carbon tab substrate (Agar Scientific, Essex, UK), a mesh cap for protection with screws (Ott *et al.*, 2008) and 150 µm conical holes and a protective holder for storing. The backing material, Teflon sheet, on the carbon tab substrate was left around the edge of the substrate to avoid the substrate sticking to the cap. One field blank was opened during mounting and dismantling (~2 × 5 min) of the UNC samplers and immediately closed during each measurement interval at each location.

#### Storage and handling of samples

The UNC samplers were mounted with carbon tabs and a mesh cap and immediately put into protective holders before the sampling event in an ISO class 6 clean room at room temperature. The UNC samplers were once again put into protective holders after the sampling was done. The UNC samplers were immediately brought back to the laboratory after the collection of particles in the working environment had been done. The samplers were kept in boxes at room temperature until analysis, which was done 3–6 months after the sample event. Each set of six UNC samplers was outside the protective holder and without the mesh cap in the SEM for 8 h of analysis and was put back with a mesh cap and in the protective holder afterwards. The analyses were made during a three-month period.

#### Microscopy, image processing, and conversion to concentration

To analyse the UNC sampler, the mesh cap was removed and the collection surface of the sampler was analysed with a SEM. Sixty images of 450 µm × 600 µm were taken, with ×500 magnification and 1.71 pixels µm^−1^ resolution, to count and size particles with a grid-like pattern making up a rectangle covering 51% of the surface projected by the circular opening of the mesh cap, with an angle-selective backscatter detector in the Carl Zeiss MERLIN FE-SEM GEMINI II (Zeiss, Germany, 2012). In addition, for one sampler from each location and time interval, in total 12 samplers, 81 images were taken with a ×3000 magnification, resolution 14.3 pixels µm^−1^, to cover the area of one image with ×500 magnification. The microscope was set to a working distance of 8.5 mm, a probe current at 300 pA, and a beam accelerating voltage at 15 kV.

The acquired images for each UNC sampler were processed in ImageJ (Version 1.48, National Institutes of Health, USA, released 2014) and MATLAB (R2014b [8.4.0.150421], The MathWorks, Inc., Natick, MA, USA, released 2014). The threshold method was primarily the RenyiEntropy method and when that did not cover the particles for the specific sampler it was changed to the triangle method. The minimum area to be considered as a particle was the area corresponding to one pixel, in our case 0.34 μm^2^ for the ×500 magnification. In addition, particles with an area larger than 10000 μm^2^ were stopped by the mesh cap. Particles touching a side of the image were excluded.

The determination of the particle mass concentration for the UNC sampler is described by Wagner and Leith (2001b) and Schneider *et al.* (2002) and the exact form we have used is found in Leith *et al*. (2007, equation 2). For the calculations of the particle mass concentrations for heterogeneous aerosols as described by Wagner and Leith (2001b) and Wagner and Macher (2003) we used the following factors for all locations: the volume shape factor 1.6 and dynamic shape factor 1.4. For the crushing station, drive station and concentrator we used the density 2.8 g cm^−3^ and for the concentrate terminal 3.75 g cm^−3^ as determined by the mining company.

To be able to compare the particle mass concentrations of each UNC sampler for PM_10_, PM_2.5_ and respirable fraction to the concentrations from an impactor and cyclone, the collection efficiency curves for each fraction from Hinds (1999) were used, as previously described by Ott and Peters (2008) and Wagner and Macher (2003). The collection efficiency curve of an impactor compared to a cyclone is different in steepness: the cyclone has a shallow curve while the impactor curve is sharp. The cut-point for the respirable fraction collection efficiency curve is at 4 µm (Hinds, 1999).

#### The mesh factor and alternative models

Wagner and Leith (2001c) introduced a mesh factor from wind tunnel experiments, $\gamma_{m}$, to account for the effects of the mesh cap on the deposition velocity. In principle, the mesh factor adapts the deposition model to align the results of the UNC sampler to the results of a cascade impactor in a wind tunnel experiment. Compared to the deposition model based solely on functions derived from physics, the model with $\gamma_{m}$ gives more weight to large particles and less to small particles. Based on our and others’ previous observations of low estimates for small particles (Shirdel *et al.*, 2017) and the fact that the wind speed was low in the present environments, an alternative expectation is that the only restriction from the mesh cap is the open area versus the closed area.

We tried two alternative analysis models (Supplementary Figure S1, available at *Annals of Work Exposures and Health* online) to Wagner and Leith’s mesh factor (2001c, equation 6). The first model, the hybrid model, modifies the current mesh factor with respect to the open area of the mesh cap (specific to size of hole diameter and pitch) by using a factor of 0.27, when $\gamma_{m}$ ≥ 0.27 [Equation (1)]. The hybrid model affects the smaller particles by introducing the primary effect of a mesh cap in the particle path,

$$\begin{array}{c} \gamma_{m}=\{\begin{array}{c} 0.27,\;\gamma_{m}\geq 0.27\\ \left(5.95\times 10^{-3}\right)\times {\left(\frac{d_{a}v_{t}}{\nu }\right)}^{-0.439},\;\;\gamma_{m}<0.27^{\prime } \end{array} \end{array}$$ (1)

where $d_{a}$ is the aerodynamic diameter of the particle, $v_{t}$ is the terminal settling velocity, and *ν* is the kinematic viscosity of air. The second model, area factor, completely replaced the mesh factor with the value of the open area, thus $\gamma_{m}$ = 0.27, taking the primary effect into account equally for all particle sizes.

### Active methods

The active sampling for PM_10_ and PM_2.5_ consisted of pre-weighed PTFE membrane filters (Zefluor, 47 mm, 2.0 µm, Pall, NY, USA), impact sampler for PM_10_ and PM_2.5_ (SKC, Inc., Eighty Four, PA, USA), respectively, and a diaphragm *pump* (Gast manufacturing, Inc., MI, USA) restricted to air flow rates of 10 L min^−1^. Correspondingly the sampling for respirable fraction consisted of a three-piece conductive polypropylene cassette (inlet section removed), a 5.0 µm polyvinyl chloride PVC 37 mm filter, an aluminium cyclone and a pump, AirChek 2000 (all SKC, Inc., Eighty Four, PA, USA), with a flow rate of 2.5 L min^−1^. Setup and sampling for the respirable fraction, as well as the weighing of the filters, including the use of an anti-static gun, followed the guidelines by NIOSH (1998). The weighing was done at room temperature with no special humidity control, as neither the filters nor the particles were considered hygroscopic. All air flows were measured at the beginning and end of each sampling period using a primary flow meter (DC-Lite, Bios International, NJ, USA) to ascertain that the air flows for PM_10_, PM_2.5_ and cyclone were within ±5% of the given flow rates.

#### Storage and analysis of samples

The filters for PM_10_, PM_2.5_ and respirable dust were weighed twice in a laboratory at room temperature before the sampling and put into protective holders. After the samples were collected, the filters were once again put into protective holders and immediately brought back to the laboratory. The filters were kept in boxes at room temperature. Two months after the samples were put into protective holders they were weighed in a laboratory at room temperature, which was repeated twice. The concentration for each filter was calculated according to the guidelines by NIOSH (1998).

### Aerodynamic particle sizer

To measure the particle size distribution a pre-calibrated APS (TSI Model 3321, range 0.5–20 µm) was used. Each sample took 20 s to collect and samples were continuously collected during each interval. The particle density 2.8 g cm^−3^ (crushing station, drive station, and concentrator) and 3.75 g cm^−3^ (concentrate terminal) were used with applied Stokes correction. The data from the APS was analysed in Aerosol Instrument Manager (version 8.1.0.0, TSI Incorporated, Shoreview, MN, USA, released 2007).

Comparison of particle size distributions between measurement instruments with different resolutions are visualised by normalising the distributions with division by the logarithmic width of each bin size. Normalisation was made by dividing the mass concentration for each bin size, dM, with the logarithm of the upper limit of the aerodynamic diameter, *d*_a_, of the bin size minus the logarithm of the lower limit of the bin size, resulting in dM/dlogd_a_, as described in Application note PR-001 from TSI.

### Blanks

There was a total of 12 field blanks for the UNC samplers, five transport blanks for the cyclones, and 9 transport blanks for the impactors. The transport blank filters for both the cyclones and impactors were kept in sealed protective holders during all times except for when they were weighed twice before and after sampling in a laboratory at room temperature. To account for contamination from manual handling the UNC sampler blanks were opened, one for each location and time, for ca. 5 min during mounting and dismantling.

For each analysis method, the mean of the UNC sampler field blanks from the crushing station, concentrator and concentrate terminal was calculated and subtracted from the UNC sampler results at those locations Supplementary Table S1, available at *Annals of Work Exposures and Health* online). A separate mean was calculated and subtracted from the results collected at the dustier drive station. Negative values due to subtraction of the mean of the field blanks from the results were not omitted or altered.

### Statistics

Firstly, descriptive statistics of particle concentrations were retrieved, including mean, max, min, standard deviation (SD), median, first quartile, third quartile and coefficient of variation (CV). *t*-Tests were used to test for statistically significant differences between mean concentrations of PM_10_ and PM_2.5_ between the UNC sampler and the impactors, and respirable fraction between the UNC sampler and the cyclone. *t*-Tests were also used to test for statistically significant differences between means for the different UNC sampler analysis models. The means for the different UNC sampler analysis models were calculated by taking the mean value of the distance squared for each UNC sampling value to the supposed true mean of the impactor or cyclone for each location and time for every particle fraction.

As an estimate of the precision and ability to discriminate between different particle concentrations in the environment, the intraclass correlation (ICC) was calculated. For each measurement device, a linear mixed effects model was fitted with particle fraction as dependent variable and location (4 levels: crushing station, drive station, concentrator, and concentrate terminal) and time (3 levels: evening, night, morning) as independent fixed effects, and a random effect allowing for different mean concentrations at each of the 12 measurement occasions. A 95% confidence interval (CI) for ICC was estimated using basic bootstrap CIs based on 1000 bootstrap samples. All statistical analyses were performed using R (R Core Team, 2016, version 3.3.2, R Foundation for Statistical Computing, Vienna, Austria, released 2016). Mixed effects models were fitted using the R package lme4 (Bates *et al.*, 2014).

The percentage of over and underestimation of the UNC sampler compared to the impactors and cyclones was calculated by fitting a linear regression model with forced intercept through zero using Origin (OriginLab, Northampton, MA, USA).

## Results

In every location, both outdoors and indoors, the wind speed was low and registered at 0 m s^−1^. The mean temperatures were −10°C (263 K) at the crushing station, 14°C (287 K) at the drive station, 19°C (292 K) at the concentrator, and 4°C (277 K) at the concentrate terminal. The relative humidity was around 20% at all of the locations except for the concentrate terminal where it was around 90%. The particles were predominantly minerals with low hygroscopicity (Supplementary Table S2, available at *Annals of Work Exposures and Health* online).

### Comparison of analysis models to other sampling methods

The crushing station had the lowest concentrations, but none of its UNC samplers had a particle count below 39 (PM_2.5_). For the other locations, no UNC sampler had a particle count below 199. When applying ×3000 magnification instead of ×500, the mean increase in mass concentration, based on nine different samplers from the indoor locations, was: 2.17 ± 0.48 times for PM_2.5_, 1.25 ± 0.35 times for respirable fraction and 1.10 ± 0.29 times for PM_10_. Acquiring one image with ×500 magnification from the SEM took 90 s, while the corresponding 81 images with ×3000 magnification took 2 h to acquire. Imaging one UNC sampler took 1.5 h with the manual SEM at ×500 magnification and would take about 120 h with ×3000 magnification with the same manual SEM analysis method. Because such long imaging time would be unrealistic in occupational hygiene applications, we chose to apply ×500 magnification even though this leads to an underestimation, most noticeable for PM_2.5_, for which the mass concentration was approximately halved.

**Figure 1.**
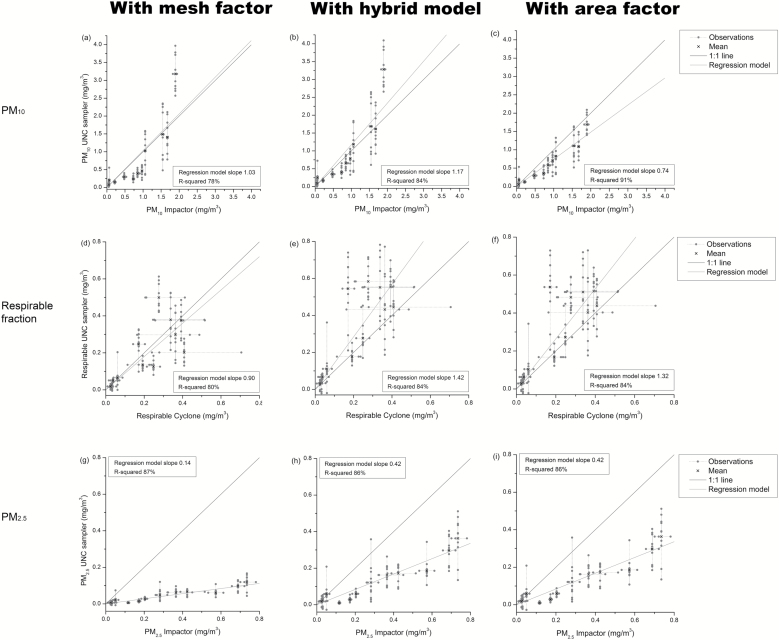
Particle mass concentrations for the UNC passive aerosol sampler versus PM_10_ impactor, respirable cyclone and PM_2.5_ impactor. The black cross is the mean for both samplers and the grey dots are each individual observation from the samplers. The value of the slope of the fitted linear regression model and *R*-squared are noted for each analysis model and particle mass concentration. (a) UNC sampler with mesh factor versus SKC impact sampler for PM_10_. (b) UNC sampler with hybrid model versus SKC impact sampler for PM_10_. (c) UNC sampler with area factor versus SKC impact sampler for PM_10_. (d) UNC sampler with mesh factor versus cyclone for respirable mass fraction. (e) UNC sampler with hybrid model versus cyclone for respirable mass fraction. (f) UNC sampler with area factor versus cyclone for respirable mass fraction. (g) UNC sampler with mesh factor versus SKC impact sampler for PM_2.5_. (h) UNC sampler with hybrid model versus SKC impact sampler for PM_2.5_. (i) UNC sampler with area factor versus SKC impact sampler for PM_2.5_.

Applying alternative analysis models for the uptake of particles altered the agreement between the UNC sampler and the other sampling methods (Fig. 1). The most noticeable difference was for the PM_2.5_ results: The UNC sampler result using the original analysis model (mesh factor) resulted in mass concentrations of 14% of the PM_2.5_ impactor results, while application of the modified analysis models gave 42%. The regression models explained the same or more of the variance for the UNC sampler with area factor compared to the other two analysis models (Fig. 1). The smallest differences between UNC sampler and impactor results were obtained using area factor (Table 1). For the cyclone, the smallest difference was obtained with mesh factor.

**Table 1.** *t*-Tests for deviation of the different UNC sampler models from impactors and cyclone: mesh factor, hybrid model, and area factor. The means tabulated for the different UNC sampler analysis models were calculated by taking the mean value of the distance squared for each UNC sampling value to the supposed true mean of the impactor or cyclone for each location and time for every particle fraction.

UNC sampler model	Mesh factor	Hybrid model	*P*-value (mesh factor versus hybrid model)	Area factor	*P*-value (mesh factor versus area factor)
Particle fraction	Deviation, mean ± SD [(mg m^−3^)^2^], 95% CI	Deviation, mean ± SD [(mg m^−3^)^2^], 95% CI		Deviation, mean ± SD [(mg m^−3^)^2^], 95% CI	
PM_10_	0.29 ± 0.63 (0.18; 0.41)	0.29 ± 0.73 (0.16; 0.42)	0.95	0.12 ± 0.20 (0.086; 0.16)	0.0050
Respirable fraction	0.014 ± 0.021 (0.010; 0.018)	0.037 ± 0.067 (0.025; 0.049)	0.00050	0.029 ± 0.058 (0.019; 0.039)	0.0093
PM_2.5_	0.11 ± 0.14 (0.087; 0.14)	0.056 ± 0.071 (0.042; 0.070)	0.00010	0.056 ± 0.071 (0.042; 0.070)	0.00010

**Figure 2.**
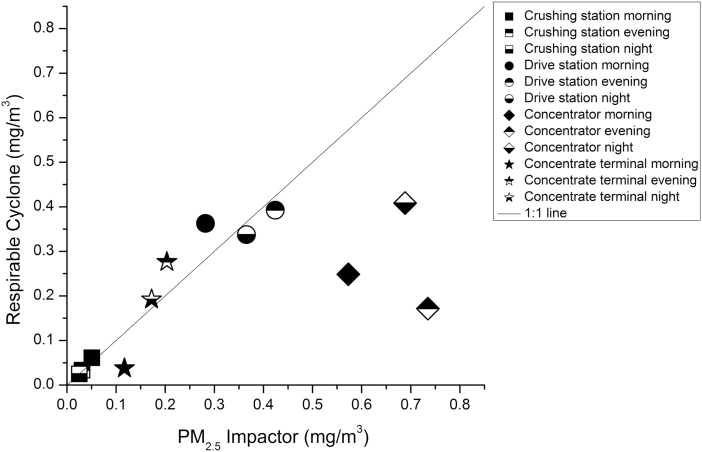
Mean particle mass concentrations at all locations for respirable cyclone versus PM_2.5_ impactor.

It is worth noting that the respirable cyclone (which has a cut-point at 4 µm, though with a different particle collection efficiency curve compared to a PM_4_ impactor) showed similar or lower results compared to the PM_2.5_ impactor (Fig. 2).

### Normalised particle size distributions

**Figure 3.**
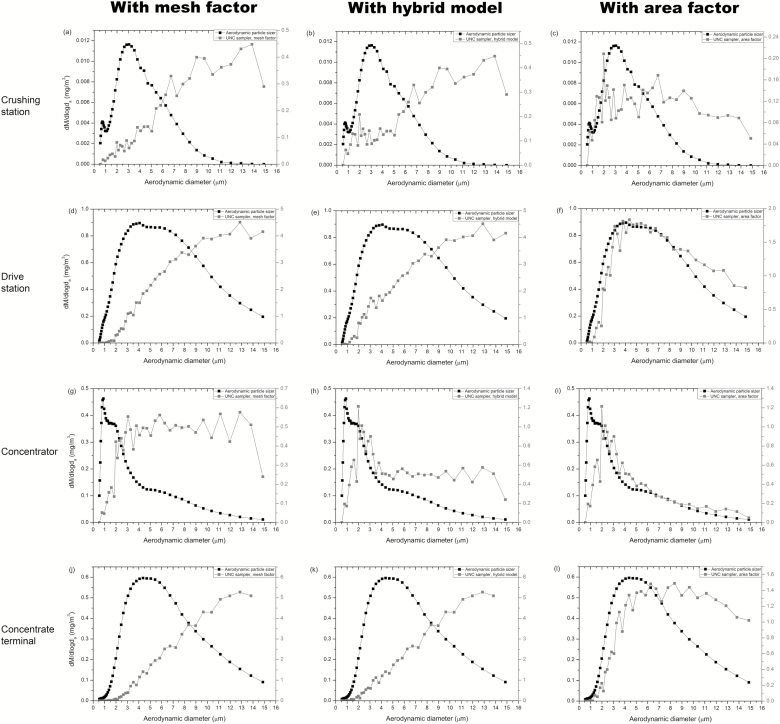
Normalised mass concentration distributions (dM/dlogd_a_) for the UNC sampler with mesh factor, hybrid model and area factor versus APS at the different locations. Crushing station: (a) mesh factor; (b) hybrid model; (c) area factor. Drive station: (d) mesh factor; (e) hybrid model; (f) area factor. Concentrator: (g) mesh factor; (h) hybrid model; (i) area factor. Concentrate terminal: (j) mesh factor; (k) hybrid model; (l) area factor. Note that the APS was not intended or calibrated for fully quantitative measurements but for characterisation of relative distributions. To best illustrate distributions we therefore used different scales (see left and right hand side of the plots) for the different samplers.

The normalised mass concentration distributions of the different UNC sampler models were depicted together with the APS data from the different locations, to compare the distributions (Fig. 3). The distributions when using the original mesh factor deviated largely from the distributions described by the APS. In contrast, the distributions when applying the area factor analysis model to the UNC sampler data showed improved agreement.

### Descriptive statistics

Focusing on model fit, the results from Table 1 and Fig. 3 indicated that the UNC sampler with area factor had an overall better agreement with the other instruments compared to the mesh factor and hybrid models. Therefore, area factor was the model we continued with for the descriptive statistics for variability (Table 2). These showed that the ICC for the UNC sampler was not as good as for the impactors, but better than the cyclone.

**Table 2.** Comparison of the variability of the different methods and descriptive statistics for data in Fig. 1, panels c, f and i. Median, first quartile (Q1), and third quartile (Q3) particle concentration for each particle size fraction for the different measuring devices at all locations. Max, min, mean, standard deviation (SD), coefficient of variation (CV), *P*-values from *t*-tests, within and between occasion variance and ICC with CIs are also noted.

Particle fraction	Measuring device	Number of samples	Median, Q1–Q3 (mg m^−3^)	Min–Max (mg m^−3^)	Mean (mg m^−3^)	SD	CV	*P*-value	Within occasion variance	Between occasion variance	ICC (95% CI)
PM_10_	UNC sampler with area factor	118	0.38 (0.13–0.85)	−0.015 to 2.1	0.57	0.52	0.92	0.058	0.045	0.17	0.79 (0.40–0.90)
	Impactor	36	0.74 (0.17–1.2)	0.044 to 1.9	0.80	0.64	1.1		0.0015	0.17	0.99 (0.97–1.00)
Respirable fraction	UNC sampler with area factor	118	0.26 (0.074–0.47)	−0.022 to 0.73	0.29	0.22	0.76	0.026	0.011	0.012	0.51 (0.11–0.74)
	Cyclone	35	0.21 (0.055–0.27)	0.0088 to 0.71	0.21	0.17	0.59		0.014	0.0043	0.24 (0–0.67)
PM_2.5_	UNC sampler with area factor	118	0.072 (0.028–0.20)	−0.027 to 0.51	0.13	0.13	1.0	0.00014	0.0039	0.0017	0.30 (0.0059–0.56)
	Impactor	36	0.24 (0.10–0.52)	0.0099 to 0.78	0.31	0.25	2.0		0.0016	0.0013	0.45 (0–0.79)

## Discussion

Initially we used the analysis model previously described by Wagner and Leith (2001a, b, c) for the particle fraction concentration calculations, applying the mesh factor. Compared to the PM_2.5_ impactor, the UNC sampler with mesh factor showed a large underestimation, as previously reported by e.g. Wagner and Macher (2003) and Shirdel *et al.* (2017). Herein, we therefore modified the analysis model of the UNC sampler and made two new analysis models. The hybrid model was still based on the original analysis model with mesh factor, but the model’s attenuation of smaller particles was limited. The area factor only considered the effect from the ratio between open and closed area of the mesh cap for all particle sizes. Both modified analysis models showed better agreement in particle concentration measurements with the PM_2.5_ impactor. The UNC sampler with area factor also showed a stronger correlation with the PM_10_ impactor (Fig. 1).

An important factor to consider is that some degree of underestimation is introduced by limitations in the microscopic resolution. There will always be a lower limit where particles will not be ‘seen’ with the UNC sampler analysis method. This limit is determined by the magnification used in the SEM and the settings in the image analysis software for detecting particles. Therefore, the smallest particles will be missed and, depending on the size distribution, the mass concentration will to some extent be underestimated. Certainly, the particle size fraction of PM_2.5_ will be most affected, which the use of different magnifications showed. For example Wagner *et al.* (2012) used a higher magnification than in the present study and reported a lower PM_2.5_ underestimation than Wagner and Macher (2003) and Shirdel *et al.* (2017). To save time and money, lower resolution is beneficial, but with the resolution used herein, analyses should be limited to PM_10_, respirable fraction and coarse fraction (PM_10-2.5_). The UNC sampler itself appears however also to be useful for PM_2.5_ if resolution is sufficient and the area factor model is used, as we observed that the remaining underestimation for the area factor model (58%, Fig. 1i) was almost completely accounted for with the higher magnification for all particle fractions. In contrast, the higher magnification still could not explain the remaining underestimation for PM_2.5_ with the mesh factor model.

The image analysis rules also introduced an underestimation, due to particles being excluded if they touch an edge of the image analysed. A rough estimate for this underestimation can be provided if we assume spherical particles. Then, given that each image was 450 µm × 600 µm, about 5% of 10 µm particles would be excluded. This underestimation will be smaller for smaller particles. Thus, the effect of this is minimal.

Another potential source of underestimation is that particles close to the hole size of the mesh cap may suffer from an interception effect. Although the holes of the UNC sampler were *ca*. 150 µm in diameter, much larger than most of the measured particles, the interception effect could be significant for particles approaching 10 µm in size, particularly if they are nonspherical.

As mentioned in the Results section, the cyclone unexpectedly estimated a lower concentration than the PM_2.5_ impactor in certain locations. A similar behaviour is apparent in the data of Wagner and Macher (2003). This could be due to measurement errors in either impactor or cyclone collection, for example an unexplained underestimation by the cyclone. However, this phenomenon is not necessarily due to an error, as a mass median aerodynamic diameter lower than 2.5 µm with a tight geometric standard deviation can result in more particles being collected by a PM_2.5_ impactor than a respirable cyclone, due to the shallowness of the cyclone’s cut-off curve. Furthermore, Chen and Huang (1999) have noted that the SKC aluminium cyclone either over or underestimates particle mass concentrations depending on the particle size distribution. Traditionally work exposure to particles is assessed by measurement of the respirable mass fraction with a cyclone. The particle distribution collected by the cyclone is however defined based on technical aspects. It was constructed to mimic human physiology, rather than defining particle distributions. In contrast, the UNC sampler can be used to describe the full particle size distribution. Any particle size cut-off, shallow or sharp, can be applied when analysing the UNC data. This is certainly an advantage with this technique.

The particle size distributions were also calculated for the different UNC analysis models. The use of the area factor model then gave very similar particle size distributions for the APS and UNC sampler. This is a significant improvement compared to the previously used analysis model (mesh factor) for the UNC passive sampler. A limitation that however should be noted is that the APS was pre-calibrated, but not specifically configured to sample isokinetically in this particular mining environment; furthermore APS counting efficiency was assumed to be constant as a function of particle size. These limitations might affect representativity as regards to coarse particles.

The mesh factor model was based on wind tunnel experiments. It may be that the mesh factor is applicable when using the UNC sampler in the outside environment where it is windy, but that it could introduce bias when it is windless, especially for small particles. This factor could to some extent be an explanation for the previously observed underestimations of small particles (Wagner and Macher, 2003; Watkins *et al.*, 2009; Shirdel *et al.*, 2017).

The area factor model used herein, only considering the ratio between open and closed area of the mesh cap, should not be interpreted as similar to the model proposed by Nash and Leith (2010) for the much smaller, mainly diffusing, ultrafine particles.

All measurements indicated low concentrations at the crushing station (Fig. 3), where it was cold and snowing. The instruments could have been affected by the snow. A layer of frost had built up on the impactors. There is a possibility that frost layers were also formed on the carbon surface of the UNC samplers and that particles were washed away when the frost melted, which could explain the difference in distributions of the UNC sampler with area factor to the APS at the crushing station.

However, none of the limitations affect the main result: Replacing the UNC sampler analysis model, from the previously used mesh factor to area factor, greatly improved the performance of the UNC passive sampler. Future studies need to be made to evaluate the area factor further, in other environments and with different particle size distributions. In addition, for future use in occupational hygiene shortening SEM analysis time is crucial. This could be achieved by e.g. applying an automated SEM or decreasing the number of images analysed.

## Conclusions

In an occupational environment with little or no wind, the UNC sampler data showed better agreement with particle mass concentrations data from impact samplers and respirable cyclones when the analysis model of the UNC sampler with mesh factor was changed to UNC sampler with area factor. In addition, the agreement with the particle size distribution, as assessed by APS, was largely improved. The precision of the UNC sampler was not as high as the impactors’, but higher than the respirable cyclone’s precision. The above results show that passive sampling can be considered an alternative to active sampling in the working environment. This opens up for further development of the UNC sampler for dust sampling in the working environment, and possibly also other passive samplers.

## Supplementary Data

Supplementary data are available at *Annals of Work Exposures and Health* online.

Supplementary Material
