# Supplementary material for: Improving the UNC Passive Aerosol Sampler Model Based on Comparison with Commonly Used Aerosol Sampling Methods
Source: Ann Work Expo Health. 2017 Dec 28;62(3):328–38. doi: 10.1093/annweh/wxx110 (PMC5873525; doi:10.1093/annweh/wxx110)
Supplement: Supplementary Material [file wxx110_suppl_supplementary-material.pdf]

Online Supplementary Material

Improving the UNC Passive Aerosol Sampler Model Based on Comparison with Commonly Used Aerosol Sampling Methods

Mariam Shirdel[1], Britt M. Andersson[2], Ingvar A. Bergdahl[1], Johan N. Sommar[1], Håkan Wingfors[3] and Ingrid E. Liljelind[1]

[1]Occupational and Environmental Medicine, Department of Public Health and Clinical Medicine, Umeå University, SE-901 87 Umeå, Sweden
[2]Department of Applied Physics and Electronics, Umeå University, SE-901 87 Umeå, Sweden
[3]Swedish Defence Research Agency, CBRN Defence & Security Division, Cementvägen 20, SE-901 82 Umeå, Sweden

Supplementary Data

Table S1 includes all means and standard deviations of the UNC sampler field blanks for the different analysis models and locations for the longest sampling time (33060 seconds).

Table S1. Mean and standard deviation (SD) of UNC sampler field blanks for mesh factor, hybrid model and area factor for the different locations.

	Mesh factor			Hybrid model			Area factor		
	PM_{10} Mean+SD (mg/m^3)	Respirable fraction Mean+SD (mg/m^3)	$PM_{2.5}$ Mean+SD (mg/m^3)	PM_{10} Mean+SD (mg/m^3)	Respirable fraction Mean+SD (mg/m^3)	$PM_{2.5}$ Mean+SD (mg/m^3)	PM_{10} Mean+SD (mg/m^3)	Respirable fraction Mean+SD (mg/m^3)	$PM_{2.5}$ Mean+SD (mg/m^3)
Crushing station, concentrator and concentrate terminal	0.019±0.012	0.0098±0.0069	0.0068±0.0050	0.036±0.024	0.027±0.019	0.023±0.017	0.032±0.022	0.026±0.019	0.023±0.017
Drive station	0.062±0.0087	0.032±0.012	0.023±0.011	0.12±0.037	0.090±0.039	0.079±0.038	0.11±0.040	0.089±0.039	0.079±0.038

X-ray diffraction (XRD) analysis was made on impactor filters, one for PM_{10} and one for $PM_{2.5}$, from each location. The amount of particles collected at the crushing station was too low for XRD readings, but the chemical compositions in weight percentage from the other locations are presented in Table S2. The particles are predominantly minerals and insoluble in water (Crawford, 2009) and will thus not absorb significant amounts of water. Adsorption of water on the surface of the particles is possible, but the effect on particle size and mass is minimal, see Ma *et al.* (2010).

Table S2. XRD analysis on two filters at each location. The chemical compositions are denoted in weight percentage (wt%).

Minerals	Drive station (wt%)	Concentrator (wt%)	Concentrate terminal (wt%)
Albite ($NaAlSi_3O_8$)	33	38	9
Bredigite ($Ca_7Mg(SiO_4)_4$)	3	7	-
Calcite ($CaCO_3$)	1	5	-
Chalcopyrite ($CuFeS_2$)	-	-	70
Microcline ($KAlSi_3O_8$)	34	27	2
Pseudowollastonite ($CaSiO_3$)	6	13	-
Pyrite (FeS_2)	-	-	13
Quartz (SiO_2)	23	12	6
Sphalerite (ZnS) and/or Pyrite (FeS_2)	1	3	-

Figure S1 shows the three analysis models for the UNC sampler with each model's respective factor, deposition velocity (terminal settling velocity multiplied with each model's factor), and particle concentration.

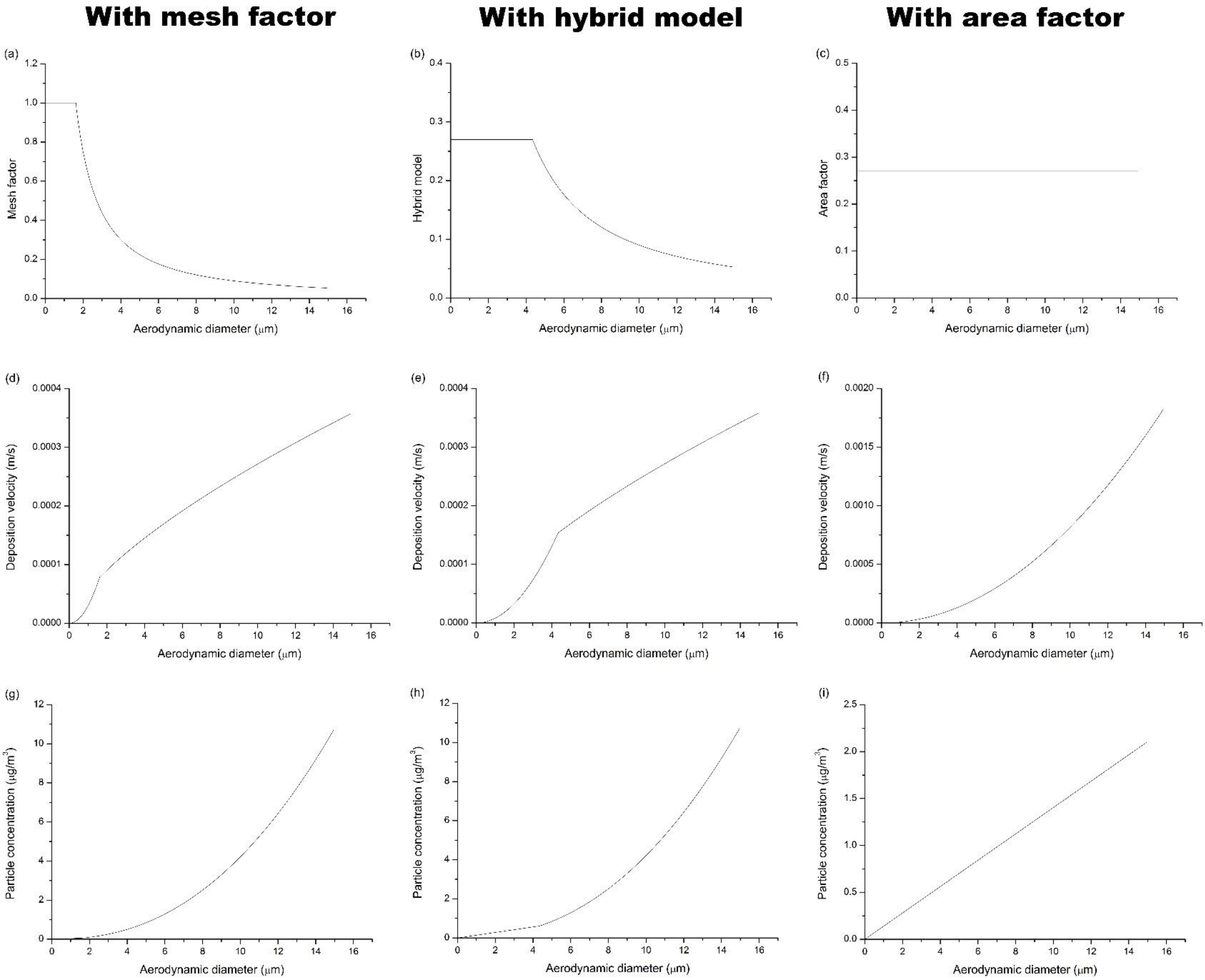

Figure S1. The three analysis models for the UNC sampler: with mesh factor, with hybrid model, and with area factor. (a) Mesh factor values for different aerodynamic diameters. (b) Hybrid model values for different aerodynamic diameters. (c) Area factor values for different aerodynamic diameters. (d) The deposition velocity with mesh factor. (e) The deposition velocity with hybrid model. (f) The deposition velocity with area factor. (g) The particle concentration with mesh factor. (h) The particle concentration with hybrid model. (i) The particle concentration with area factor.

References

Crawford J. (2009) Solubility Data on 646 Common and Not So Common Minerals [internet]. Website last updated 6 Mars 2009. Available online: https://www.mindat.org/article.php/553/Solubility+Data+on+646+Common+and+Not+So+Common+Minerals (accessed on 20 October 2017).

Ma, Q. X., He, H., and Liu, Y. C. (2010) In situ DRIFTS study of hygroscopic behavior of mineral aerosol. Journal of Environmental Sciences; 22: 555–560.
